# A *6.5kb* Intergenic Structural Variation Exacerbates the Fitness Cost of P450-Based Metabolic Resistance in the Major African Malaria Vector *Anopheles funestus*

**DOI:** 10.3390/genes13040626

**Published:** 2022-04-01

**Authors:** Magellan Tchouakui, Leon M. J. Mugenzi, Murielle J. Wondji, Micareme Tchoupo, Flobert Njiokou, Charles S. Wondji

**Affiliations:** 1 Centre for Research in Infectious Diseases, Yaoundé P.O. Box 13591, Cameroon; leon.mugenzi@crid-cam.net (L.M.J.M.); murielle.wondji@lstmed.ac.uk (M.J.W.); micareme.tchoupo@crid-cam.net (M.T.); 2 Parasitology and Ecology Laboratory, Department of Animal Biology and Physiology, Faculty of Science, University of Yaoundé 1, Yaoundé P.O. Box 812, Cameroon; njiokouf@yahoo.com; 3 Department of Biochemistry and Molecular Biology, Faculty of Science University of Buea, Buea P.O. Box 63, Cameroon; 4 Department of Vector Biology, Liverpool School of Tropical Medicine, Pembroke Place, Liverpool L3 5QA, UK

**Keywords:** malaria, *Anopheles funestus*, vector control, metabolic resistance, cytochrome P450, fitness cost

## Abstract

Metabolic-based resistance to insecticides limit the control of medically important pests, and it is extremely detrimental in the ongoing struggle to control disease vectors. Elucidating the fitness cost of metabolic resistance in major malaria vectors is vital for successful resistance management. We established the fitness cost of the *6.5kb* structural variant (*6.5kb-sv*) between the duplicated *CYP6P9a/b* P450s using the hybrid strain generated from the crossing between two *An. funestus* laboratory strains. Furthermore, we assessed the cumulative impact of this marker with the duplicated P450 genes. We established that individuals that were homozygote for the resistant structural variant (SV) presented reduced fecundity and slow development relative to those that were homozygote for the susceptible SV. Furthermore, we observed that *6.5kb* act additively with *CYP6P9a* and *CYP6P9b* to exacerbate the reduced fecundity and the increased development time of resistant mosquitoes since double/triple homozygote susceptible (SS/SS/SS) significantly laid more eggs and developed faster than other genotypes. Moreover, a restoration of susceptibility was noted over 10 generations in the insecticide-free environment with an increased proportion of susceptible individuals. This study highlights the negative impact of multiple P450-based resistance on the key physiological traits of malaria vectors. Such high fitness costs suggest that in the absence of selection pressure, the resistant individuals will be outcompeted in the field. Therefore, this should encourage future strategies based on the rotation of insecticides to reduce selection pressure and to slow the spread of pyrethroid resistance.

## 1. Introduction

Mosquitoes transmitting borne disease are one of the deadliest animals in the world. Their ability to transmit the diseases to humans causes nearly a million deaths every year, mainly in Africa [1,2]. A range of diseases including malaria are transmitted to humans by mosquitoes and more than half of the world’s population lives in areas where these mosquitoes are present [1]. Sustained mosquito control efforts are important to prevent outbreaks of these diseases. Malaria prevention in Africa, for example, relies extensively on mosquito control using pyrethroid-based, insecticide-treated bednets [3,4]. Pyrethroid is the most used insecticide class for bednet impregnation due to its low toxicity for humans and its fast-acting property [5]. This insecticide which comprises type I (Permethrin) and type II (deltamethrin, alphacypermethrin, etc.) targets the voltage-gated sodium channels where binding to sodium channels causes excitatory paralysis of the insects, followed by death [6]. Pyrethroids are extensively used for controlling insect pests in agriculture, public health, and animal health [7]. The scale-up of these pyrethroid based-tools has significantly contributed to the important reduction of the malaria burden in the past decade [3,8]. However, the increasing resistance of major vectors to current-generation insecticides and shifting vector behaviour toward increased outdoor biting (thus avoiding insecticide-treated surfaces) is a serious challenge to these vector control interventions [2,9]. Developing novel interventions and ensuring their sustainability is of great importance due to widespread insecticide resistance to WHO-approved insecticides used for vector control [10].

In response to this challenge, the Global Plan for Insecticide Resistance Management (GPIRM) was launched in 2012 to create strategies to preserve the effectiveness of current vector control tools. To significantly reduce malaria morbidity and mortalities, the WHO developed at the same time new and innovative vector control tools for the future [11]. Unfortunately, the emergence of insecticide resistance not only shortens the lifespan of the existing vector control tools but also undermines the efficacy of novel developed vector control products through cross-resistance or multiple resistance [12]. In the face of the increasing reported cases of cross-resistance or multiple resistance to pyrethroids in major malaria vectors, it is imperative to implement suitable insecticide resistance management (IRM) strategies to reduce the negative impact of such resistance. Knowledge of the molecular changes and the evolutionary process driving insecticide resistance in mosquito populations and the associated fitness costs is crucial for more effective resistance management [2].

Insecticide resistant mosquitoes usually have an adaptive advantage compared to those that are susceptible in insecticide selection pressure. In this case, the frequency of this resistant allele or the resistant profile of the population would decrease over time in the absence of treatments although the costs of resistance could be mitigated or even eliminated by compensatory mechanisms called modifiers, which are mainly selected by continued use of an insecticide following the emergence of resistance [13]. The extent to which a resistant population experiences a different environment with or without treatment may have a strong influence on the frequency of resistance alleles and the rate at which they change. One of the current and the most effective insecticide-resistant management strategies is the rotation of insecticides of different modes of action to retard or to reverse the spread of resistance [2], though it is not clear how frequently the insecticides should be rotated. Understanding the fitness cost of insecticide resistance in mosquitoes and tracking changes in resistance mechanisms in different environments and species over time will greatly improve resistance management strategies.

To date, only a few studies have measured the effect of continuous exposure to pyrethroids on the key life traits of mosquitoes and evaluated how resistance mechanisms evolve and/or regress upon the removal or the reduction of the insecticide. One of the key challenges of assessing the fitness costs of such resistance (particularly metabolic resistance, which is the most common) is due to the lack of molecular markers in major malaria vectors such as *An. funestus*. So far, the resistance to pyrethroids reported in malaria vectors is driven mostly by two major documented resistance mechanisms: (a) metabolic detoxification degrading the insecticide before it reaches the target site and (b) target-site insensitivity, which is a single nucleotide polymorphism affecting the voltage-gated sodium channel targeted by insecticides [14]. Metabolic detoxification has majorly been linked to elevated levels of P450 monooxygenase and glutathione S-transferase enzymes [8,15], but little is known about the underlying mechanisms or how these modifications affect the life traits of the vector. The design of the DNA-based diagnostic tools for target-site resistance since the late 1990s [16,17] allowed for the evaluation of the fitness cost due to target-site resistance on the different physiological traits of mosquitoes from various species [18,19,20,21,22]. However, the fitness cost associated with metabolic resistance, a very common resistance mechanism [23], remains poorly characterized in *An. funestus*. Some studies have been done on *Aedes aegypti* showing that both *kdr* and CYP-mediated resistance induced a fitness cost affecting various physiological traits of the vector, including adult longevity and mating competition [24]. It was also shown in Drosophila using a CRISPR/Cas9 genome modification approach that combinations of the *kdr* target site and *Cyp9J28* (P450-based resistance) had a fitness disadvantage with regard to development time and the fecundity of the flies, showing that these genes could be unfavorable in field populations in the absence of insecticide selection pressure.

Significant progress has been made in *An. funestus* by detecting molecular markers for cytochrome P450-based resistance. This involves mainly the cis-regulatory variants driving the expression of the duplicated pyrethroid resistance genes *CYP6P9a* and *CYP6P9b* in this species [25,26]. Two novel PCR diagnostic assays were further designed to allow the detection and the monitoring of these mechanisms as well as assessing the associated fitness cost [25,26]. Furthermore, a major structural variation (SV) in the shape of a *6.5kb* insertion (Figure S1) was reported in pyrethroid resistance *An. funestus* in the intergenic regions of the two genes (*CYP6P9a* and *CYP6P9b*) [27]. The study demonstrated that this structural variation is playing an enhancer role in the regulation of *CYP6P9a* and *CYP6P9b* expression in *An. Funestus,* raising the prospect that such structural variation could exacerbate the fitness costs of these genes due to a high energetic cost. Establishing the potential influence of such structural variations and the combined effect with *CYP6P9a* and *CYP6P9b* could help to establish the scale of fitness cost due to multiple P450-based resistance and to design better insecticide resistance management strategies.

This study sought therefore to explore the fitness costs associated firstly with the *6.5kb-SV* before assessing the interaction between this variant and each of the two genes (*CYP6P9b* and *CYP6P9a*) and to also evaluate the cumulative effect of the three resistance mechanisms on some key life trait of *An. funestus.* On the other hand, the changes in insecticide resistance mechanisms over 10 generations in the absence of selection pressure were monitored.

## 2. Materials and Methods

### 2.1. Mosquito Strains

The study was carried out with a hybrid strain generated from reciprocal crossing between the highly pyrethroid-resistant strain FUMOZ-R (*CYP6P9a/b/6.5kb-SV_R*) and the fully susceptible FANG strain (*CYP6P9a/b/6.5kb-SV_S*). Once the first generation of the crossing was obtained (F_1_), the hybrid strain was reared to the F_10_ generation. The frequency of the resistant alleles was monitored in each generation for the potential restoration of susceptibility and the fitness cost was evaluated using the F_4_/F_8_ generation.

### 2.2. Life Trait Experiments

The fitness cost associated with the SV was evaluated by comparing the reproductive parameters (female fecundity/fertility, larval development time, and female adult longevity) between a mosquito’s homozygote resistance for the SV and heterozygote and homozygote susceptible individuals for the SV. The effect of this marker on female fecundity was assessed by comparing the egg-laying ability, the median number of eggs laid, and the hatch rate between different genotypes as previously described by Tchouakui et al. [28]. To assess the combined effect of the SV and the duplicated *CYP6P9a/b* on mosquito fitness, we compared the parameters above between triple homozygote resistant and triple homozygote susceptible.

Two days post-hatching, after recording the total number of larvae produced per female, all larvae from the three genotypes for each marker were pooled and reared together in the same larvae bowl under the same environmental conditions. A set of approximately 100 larvae at different stages (L1, L2, L3, and L4) was genotyped to estimate changes in genotype frequency during the development and to indirectly measure the time of development of the immature stage, including larval mortality rates. The dynamic of pupae formation was evaluated by comparing the genotype and the allele frequencies from the starting of pupation to the end.

Approximately 100 mosquitoes were genotyped for each of the three mechanisms at D1, D10, D20, and D30 post-emergence of adults. The lifespan of homozygous resistant adult mosquitoes was compared to that of susceptible and heterozygote mosquitoes by assessing the frequency of *CYP6P9a/CYP6P9b/6.5Kb-SV* genotypes or alleles at different time points (D1, D10, D20, and D30).

### 2.3. Restoration of Susceptibility in the Absence of Insecticide

Cage experiments were used to evaluate the dynamics of *CYP6P9a*_R, *CYP6P9b*_R, and *6.5kb-SV_*R resistant allele frequencies in the absence of insecticide pressure. The progeny obtained from crosses between female FANG and male FUMOZ were let in cages for intercrosses for ten generations. In the first generation, the frequency of the *CYP6P9a*_R, *CYP6P9b*_R, and *6.5kb-SV_*R resistant alleles was assessed and then monitored in the following generations by genotyping a set of females aged between 2–5 days old. Each generation consisted of approximately 3 cages of at least 200 mosquitoes/cage of all genotypes.

### 2.4. Genotyping of Resistance Markers

The LIVAK method was used to extract genomic DNA from all larval and pupal stages as well as the adult mosquitoes [29]. The genotyping of the *CYP6P9a* resistance allele was done using PCR restriction fragment length polymorphism (RFLP) (using a *Taq*1 restriction enzyme) method as recently described [25]. As described previously [26], *CYP6P9b*-mediated resistance was also genotyped by PCR-RFLP. The restriction enzyme for this marker was *NmuC*l (*Tsp*45I) (cut site 5′-GTSAC-3′). This restriction enzyme cuts the susceptible mosquitoes in two fragments of 400 bp and 150 bp, whereas the resistant is uncut at 550 bp. For the *6.5kb-SV*, a multiplex PCR using three primers, two (FG_5F: CTT CAC GTC AAA GTC CGT AT and FG_3R: TTT CGG AAA ACA TCC TCA A) at the region flanking the insertion point, and a third primer (FZ_INS5R: ATATGCCACGAAGGAAGCAG) in the *6.5kb* insertion was designed for genotyping [27]. The amount of 1U KAPA Taq polymerase (Kapa Biosystems, Boston, MA, USA) in 1× buffer A, 25 mM MgCl_2_, 25 mM dNTPs, 10 mM of each primer was used to constitute a 15 µL PCR reaction mix using the following conditions: an initial denaturation step of 3 min at 95 °C, followed by 35 cycles of 30 s at 94 °C, 30 s at 58 °C, and 60 s at 72 °C, with 10 min at 72 °C for a final extension. The amplicon was revolved on a 1.5% agarose gel stained with Midori Green Advance DNA Stain (Nippon genetics Europe GmbH), and it was revealed on a UV trans-illuminator. Samples without an insertion are expected to give a band at 266 bp, while samples with an insertion are expected to give a band at 596 bp.

## 3. Results

### 3.1. Effect of the 6.5kb-SV on Female Fecundity

**Figure 1:**
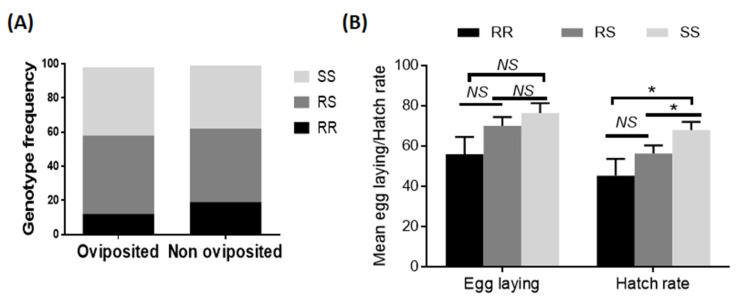
Influence of the *6.5kb* structural variant on the life-traits of *Anopheles funestus*: (**A**) contingency graph showing the proportion of each genotype at the *6.5kb-sv* locus in the oviposited females compared to the non-oviposited group, (**B**) comparison of mean number of eggs laid and mean hatch rate between genotypes. *NS* = no significant; * significant difference at *p* < 0.05.

After oviposition and genotyping of females that laid eggs and those that did not lay eggs, no significant difference (*χ*^2^ = 3.8; *p* = 0.1) was observed in the overall distribution of genotypes between oviposited females and the non-oviposed (Figure 1A), although females with the *6.5kb-SV*_SS genotype tended to lay more eggs (76.4 ± 4.8) than *6.5kb-SV*_RR (55.9 ± 5.5) and *6.5kb-SV*_RS (69.9 ± 4.4) (Figure 1B). Concerning the viability of eggs laid, the mean hatch rate of *6.5kb-SV*_SS (67.9%) was higher compared to *6.5kb-SV*_RR (45.2%) (*p* = 0.005) and *6.5kb-SV*_RS (56.3%) (*p* = 0.02) (Figure 1B).

### 3.2. Cumulative Effect of 6.5Kb-SV and the Duplicated CYP6P9a and CYP6P9b on the Fecundity of Female An. funestus

**Figure 2:**
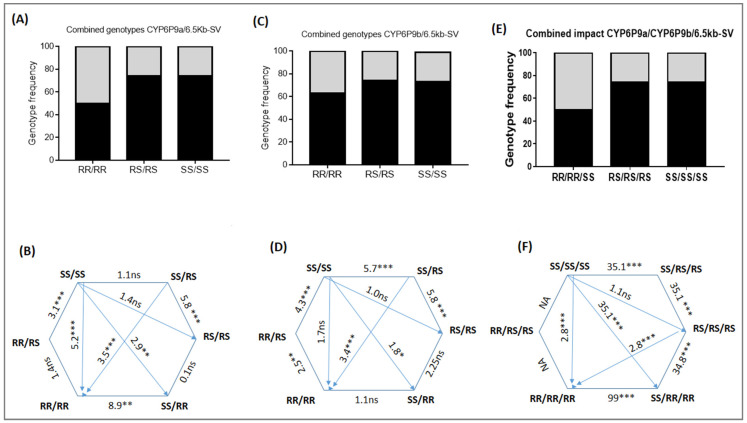
Cumulative effect of *CYP6P9a*, *CYP6P9b*, and *6,5kb-SV* on the ability of female *An. funestus* to lay eggs. Upper figures (**A**,**C**,**E**) represent the distribution of predominant genotype combinations between oviposited (black) and non-oviposited females (grey); (**B**,**D**,**F**) represent the combined effect of the three mechanisms *CYP6P9a*, *Cyp6p9b*, and *6,5kb-SV* on laying success with odd ratio (OR); numerical values indicate the OR, * level of significance (* *p* < 0.5, ** *p* < 0.01, *** *p* < 0.001); *NS*: not significant; NA represents cases where OR were not evaluated because of the low sample size.

Several combinations of genotypes were observed, including RR/RR, RR/RS, RR/SS, RS/RS, SS/RS, and SS/SS in the group of mosquitoes that laid eggs and those non-oviposited (Figure 2). A comparison of the distribution of both sets of genotypes revealed that mosquitoes that were double homozygote resistant (RR/RR) at the *CYP6P9a* and the *6.5kb-SV* genes had by far a significantly lower ability to lay eggs than RS/RS and SS/SS (Figure 2A,B), showing that both loci act additively to influence the fecundity of females when mosquitoes are double homozygote resistant. The same trend was observed when combining *CYP6P9b* and *6.5kb-SV*, although the effect was less pronounced in this last case (Figure 2C,D). Mosquitoes with the RR/RR genotype had less of a chance of oviposition compared to SS/SS (OR = 5.2; CI = 2.7–9.9; *p* ˂ 0.0001), RS/RS (OR = 2.4; CI = 1.3–6.4; *p* = 0.001), and RR/SS (OR = 8.9; CI = 4.2–18.8; *p* ˂ 0.0001), whereas the difference was not significant against RR/RS (OR = 1.4; CI = 0.8–2.5; *p* = 0.1) (Figure 2B,C).

**Figure 3:**
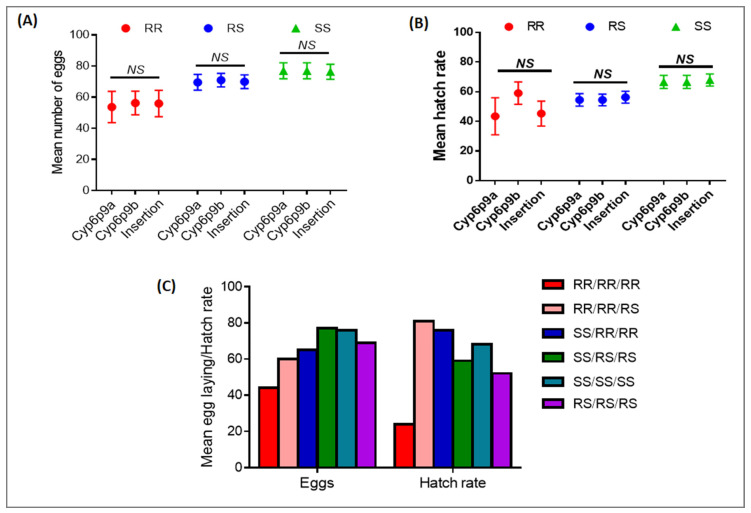
Combined impact of *CYP6P9a*, *CYP6P9b,* and *6.5kb-SV* on fecundity and fertility. Comparison of the number of eggs laid (**A**) and the hatch rate (**B**) between the same genotype for the three markers; (**C**) combined impact of *CYP6P9a*, *CYP6P9b*, and *6.5kb-SV* on the egg laying and hatching rate; *NS*: not significant.

On the other hand, SS/SS/SS triple homozygote mosquitoes had more of a chance of laying eggs compared to RR/RR/RR (OR = 2.8; *p* = 0.0003), although lesser than SS/SS vs. RR/RR probably because of the low sample size. The same pattern was noticed when comparing to SS/RS/RS and SS/RR/RR (OR = 35.1; *p* ˂ 0.0001). When compared to RR/RR/RR (OR = 2.8; *p* = 0.0003), RS/RS/RS also had more of a chance of laying eggs (Figure 3B,C). Moreover, SS/RS/RS also had more chances of laying eggs compared to RR/RR/RR (OR = 99; *p* ˂ 0.0001) (Figure 3B,C).

However, no difference was found in terms of the number of eggs laid by females with RR genotypes for the SV or *CYP6P9a/b* compared to the RS and the SS counterparts (Figure 3A,B). Yet, when combining all three markers, mosquitoes with RR/RR/RR genotypes laid fewer eggs than RS/RS/RS (*t* = 2.2, *p* = 0.02) and SS/SS/SS (*t* = 2.8, *p* = 0.008). The hatch rate of RR/RR/RR was also lower compared to that of RS/RS/RS (*t* = 1.9, *p* = 0.003) and SS/SS/SS (*t* = 3.2, *p* = 0.005) (Figure 3C). All of these results show that the *6.5kb-SV* reduces the fecundity of the female *An. funestus* and this is exacerbated when combining with the duplicated *CYP6P9a* and *CYP6P9b*.

### 3.3. Influence of the 6.5Kb-SV on Larval Development and Mortality and Pupae Formation

**Figure 4:**
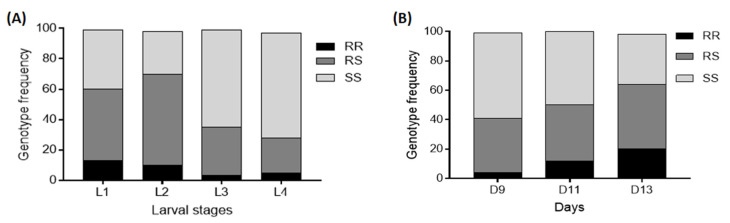
Influence of the *6.5kb* structural variant on the larval development and pupae formation in *An. funestus*: (**A**,**B**) contingency graphs showing the proportion of various genotypes at the *6.5kb-sv* locus at each larval developmental stage and pupae formation time, respectively.

After recording the total number of larvae produced per female two days post-hatching, all larvae from the three genotypes for each marker were pooled and reared together. A set of approximately 100 larvae at different stages (L1, L2, L3, and L4) was then genotyped to estimate changes in genotype frequency during the development and to indirectly measure the time of development of the immature stage, including larval mortality rates. Genotyping results revealed that the proportion of homozygote resistant larvae for the *6.5kb-SV* decreased from L1 (11%) to L4 (5%) larval stages but because of the low number of mosquitoes with the RR genotype, the difference was not significant (χ^2^ = 1.7; *p* = 0.2) (Figure 4A). For the heterozygote *6.5kb-SV_RS* genotype, a significant decrease was observed from L1 (49%) to L4 (27%) (χ^2^ = 7.2; *p* = 0.007). The significantly increased proportion of the homozygote susceptible genotype *6.5kb-SV_SS* from L1 (40%) to L4 (68%) (*χ*^2^ = 12.15; *p* = 0.0004) confirmed that mosquitoes with this genotype survived better (Figure 4A). This highlights a significant fitness cost of this structural variant on the larval development of resistant mosquitoes.

The dynamic of pupae formation was evaluated by comparing the genotype and allele frequencies from the start of pupation until the end. Genotyping of approximately 100 pupae for each of the three mechanisms at D9, D11, and D13 post-hatching revealed a consistent decrease in the proportion of the homozygote susceptible *6.5kb-SV_SS* genotype. The proportion of 58% for *6.5kb-SV_SS* larvae obtained in D9 post-hatching decreased to 34% (*χ*^2^ = 1.73; *p* = 0.19) in D11 together with a significantly increase of the frequency of homozygote resistant genotype *6.5kb-SV_RR* and heterozygote *6.5kb-SV_RS* from D9 to D13 (*χ*^2^ = 11.17; *p* = 0.0008). This confirms that homozygote susceptible mosquitoes developed significantly faster than homozygote resistant and heterozygote mosquitoes (Figure 4B). This was confirmed by evaluation of the odds ratio showing that SS have a greater chance of developing compared to RS (OR = 2.0; CI = 1.1–3.7; *p* = 0.01) and RR (OR = 3.1; CI = 1.0–9.9; *p* = 0.003).

**Figure 5:**
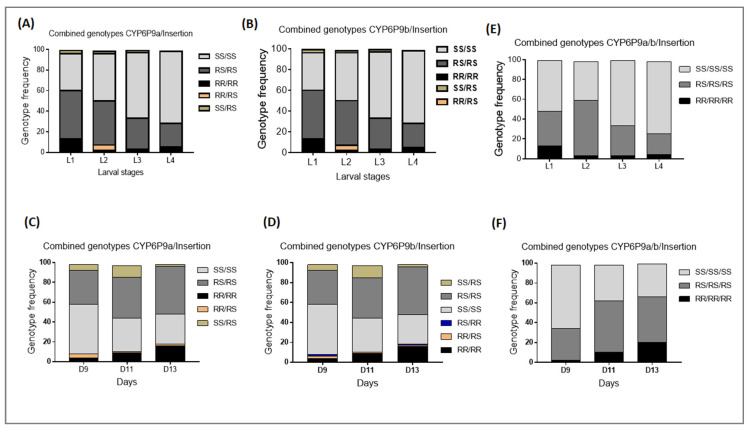
Cumulative impact of *CYP6P9a*, *CYP6P9b,* and *6.5kb-SV* on larval mortality and pupae formation. (**A**,**B**,**E**) combined impact of different markers on larval mortality, (**C**,**D**,**F**) combined impact of *CYP6P9a/b* and insertion on the kinetic of pupae formation; L1, L2, L3, and L4 represent the four larval stages whereas D9, D11, and D13 represent the number of days post-hatching where pupae were genotyped.

When combining this *6.5kb-sv* with both *CYP6P9a* and *CYP6P9b*, the effect on larval development and pupae formation was exacerbated (Figure 5A,B). The survival of SS/SS was better than RS/RS and RR/RR, and SS/SS/SS also survived better than RS/RS/RS and RR/RR/RR, as the proportion of susceptible double/triple homozygotes increased significantly during the larval stages (Figure 5E). Additionally, SS/SS developed significantly faster than RR/RR (OR = 9.3; CI = 2.9–29.9; *p* ˂ 0.0001) and RS/RS (OR = 2.9; CI = 1.5–5.4; *p* = 0.003) (Figure 5C,D). Furthermore, a significant decrease in the proportion of SS/SS/SS mosquitoes was observed from D9 to D13 (*χ*^2^ = 7.7; *p* = 0.005) together with an increase in the increased proportion of RS/RS/RS (*χ*^2^ = 2.9; *p* = 0.08) and RR/RR/RR/(*χ*^2^ = 3.6; *p* = 0.05) (Figure 5F).

### 3.4. Assessment of the Association between 6.5kb-sv Resistant Allele and Adult Longevity

**Figure 6:**
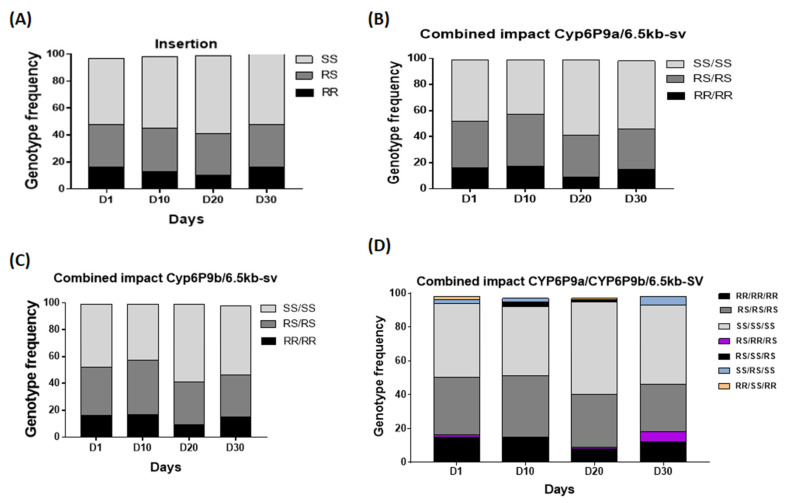
Cumulative effect of *CYP6P9a*, *CYP6P9b*, and *6.5kb-SV* on adult longevity. Effect of the *6.5kb-sv* on adult longevity (**A**), Combined impact of *6.5kb-SV* with *CYP6P9a* (**B**) and *CYP6P9b* (**C**) and combined impact of both CYP6P9a and *CYP6P9b* with *6.5kb-SV* on adult longevity (**D**); D1, D10, D20, and D30 represent the number of days post-emergence where the adults were genotyped.

After the emergence of adults, approximately 100 mosquitoes were genotyped for each of the three mechanisms at D1, D10, D20, and D30. The lifespan of homozygous resistant adult mosquitoes was compared to that of susceptible and heterozygote mosquitoes by assessing the frequency of *CYP6P9a/CYP6P9b/6.5Kb-SV* genotypes or alleles at the different time points (D1, D10, D20, and D30). As previously observed for the duplicated *CYP6P9a* and *CYP6P9b*, no significant difference was observed in the longevity of *6.5kb-sv*-resistant mosquitoes compared to those that were susceptible after genotyping of 100 alive mosquitoes at D1, D10, D20, and D30. Evaluation of the association between the *6.5kb-sv*-R allele and adult longevity and comparison of genotypes frequency showed no difference in the distribution of genotypes (*χ*^2^ = 2.7; *p* = 0.8) at these time points. Even when combining the *6.5kb-SV* with *CYP6P9a* or *CYP6P9b*, the distribution of genotypes was still not different (*χ*^2^ = 6.9; *p* = 0.3) (Figure 6A,B). This was the case also when combining all of the three markers (*χ*^2^ = 5.7; *p* = 0.4) (Figure 6C).

### 3.5. Assessment of the Restoration of Susceptibility

**Figure 7:**
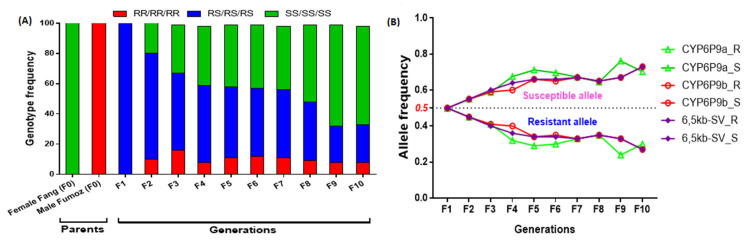
Evaluation of the reversal to susceptibility in the hybrid colony Fang/Fumoz: Changes in the *CYP6P9a*, *CYP6P9b,* and *6.5kb-SV* genotypes (**A**) and allele (**B**) for ten generations in the insecticide-free environment; F represents each generation; the dotted line indicates a frequency of 50% for the resistant and the susceptible alleles.

Restoration of susceptibility was assessed by examining changes in the frequency of the *6.5kb-SV_R* allele over 10 generations in an insecticide-free environment. In the F_1_ generation, a 50% frequency of the *6.5kb-SV_R* resistant allele was obtained since the parental lines were fully homozygotes (resistant: FUMOZ, Susceptible: FANG) for this insertion. Through generations, a significant and a consistent increase in the proportion of homozygote susceptible mosquitoes was observed from F_2_ (20%) to F_10_ (58.1%) (*χ*^2^ = 14.1; *p* = 0.002) (Figure 7A; Table S1) as well as the frequency of the resistant allele (*χ*^2^ = 4.8; *p* = 0.02) (Figure 7B; Table S1). The same pattern was also observed for all of the two duplicated *CYP6P9a* or *CYP6P9b* (Figure 7). The effect was exacerbated when combining the three markers since the SS/SS/SS genotype increased in proportion (*χ*^2^ = 17.9; *p* ˂ 0.0001) (Figure 6C).

## 4. Discussion

In the absence of insecticide application, resistant alleles could induce energetic costs or fitness disadvantages in comparison with those that were susceptible. In this study, we evaluated the fitness cost of the *6.5kb* intergenic insertion between two duplicated P450 (*CYP6P9a* and *CYP6P9b)* in the malaria vector *An. funestus*. We further established the cumulative effect of this structural variant with the duplicated P450-based resistance on various life-traits of *An. funestus*. This revealed a significant cost linked with the insertion and that this structural variation combines with *CYP6P9a* and *CYP6P9a* to additively exacerbate the reduced fecundity and the increased development time of resistant mosquitoes leading to restoration of susceptibility in the absence of selection pressure.

### 4.1. The 6.5kb Structural Variant Affects the Fecundity/Fertility of Female An. funestus

The *6.5kb-SV* induced a reduction in mosquitoes’ fecundity and fertility as previously observed for *CYP6P9a* and *CYP6P9b* [28]. Mosquitoes that were homozygous resistant for the insertion had a lesser chance of laying eggs compared to the homozygote susceptible as their proportion was among those that laid eggs. The effect was exacerbated when mosquitoes were double/triple homozygote resistant for the three markers as mosquitoes with the RR/RR/RR genotype showed a significantly reduced number of eggs and a hatch rate compared to females with the SS/SS/SS genotype. The number of eggs laid by females directly relates to the amount of ingested blood. For example, a 15% reduction in the amount of ingested blood was observed in the temephos resistant females of *A. aegypti* as compared to their susceptible control and as a result, they deposited 21% fewer eggs [30]. In our results, we noticed a reduction in both the fecundity and the hatchability of the resistant compared to their susceptible counterparts. Also, the ability of mosquitoes with resistant genotypes to lay eggs was lesser than that of mosquitoes with the susceptible genotype. In contrast to this observation, a study conducted on the same strains of *An. funestus* showed some reproductive advantages in resistant individuals as compared to the susceptible control [31]. In this study, because of the lack of a molecular marker for metabolic resistance, the authors compared only the reproductive parameters of resistant strains to that of the susceptible without crossing. Mebrahtu et al. [32] observed in contrast that the rate of inseminated *Ae. aegypti* females from a field population resistant to permethrin was lower when compared to a susceptible field strain. Additionally, resistant females took longer to lay their eggs. Lower insemination rates observed for γ-HCH and dieldrin resistant *An. gambiae* and *An. stephensi* females were attributed to lower activity in resistant males [33]. These two factors could explain the difference observed in our study. Similar results were obtained also in *Cx. pipiens pallens* where the proportion of laid and viable eggs by Gelugor females was less than the two other susceptible strains, which can be linked to the low insemination rate of resistant females. In *Culex* [34] and *Aedes* [22] *mosquitoes,* the pyrethroid resistant strain displayed a reduction of approximately 30% in viable eggs. Tchouakui et al. (2018) reported recently the same reduction of the fecundity of female *An. funestus* caused by metabolic based-resistance using the L119F-GSTe2 marker [35]. This is the first study evaluating the fitness cost of the *6.5kb* structural variant and an association with multiple P450 genes. As observed in this study, the SV combined with *CYP6P9b* beside *CYP6P9a* to influence the fecundity of *An. Funestus*. In the previous study with an experimental hut assessing the impact of SV beside *CYP6P9b* and *CYP6P9a,* it was noticed that the SV alone does independently reduce the efficacy of bed nets particularly pyrethroid-only nets beside the duplicated *CYP6P9b* and *CYP6P9a* [25]. In the same study, a greater reduction of bed net efficacy was observed when the SV was combined with both *CYP6P9b* and *CYP6P9a* since double/triple homozygote resistant mosquitoes were far more able to survive exposure to pyrethroid-only nets than all genotypes. Such an additive burden of the duplicated cytochrome P450s indicates the greater risk that metabolic resistance poses to insecticide-based interventions. Rotation of the insecticide, could help to reduce such a negative impact of multiple resistance because of the high associated fitness cost. However, resistance management strategies should be implemented before the resistance alleles become fixed in field populations as, it is now unfortunately the case in some parts of southern Africa, such as southern Mozambique [36].

### 4.2. Effect of the 6.5kb-SV_R Resistant Allele on the Developmental Time of the Larvae

In this study, We observed a greater mortality/slower development of the resistant mosquitoes during larval development compared to the susceptible, as previously observed for the *CYP6P9a* [28]. The overproduction of these detoxifying enzymes requires that a significant part of insect energy resources be redirected to the machinery related to the metabolism and the excretion of the xenobiotic, such as insecticides [37]. This energy trade-off is sustained at the expense of other physiological aspects of the organism, which may reflect negatively on the basic demands of its biology [38]. In particular, this is expected when metabolic resistance is the mechanism involved and the effect is probably exacerbated in the presence of multiple P450s. This situation, which has a strong potential impact on vector viability, may have occurred in the *An. funestus* strain used in this study, showing clear evidence of the fitness cost imposed by P450-based metabolic resistance in mosquitoes. Probably the over-expression of *CYP6P9a/b* enhanced by the SV insertion results in decreased locomotive performance and less competitiveness for food, limiting the ability of mosquitoes with the resistant allele to move faster to feed as previously observed in resistant *Culex pipiens* for carboxylesterase-mediated metabolic resistance [39]. Such an increase in the development time of resistant mosquitoes is good news for IRM since the dissemination of mosquito populations in the field mainly depends on the developmental time [22]. In fact, in the natural environment the dynamics of population development, such as a prolonged larval stage, may affect the adaptive advantages of an individual due to several extrinsic factors, such as the elimination of breeding habitats [40], the presence of predators or parasites, which can reduce the larval survival rate [41] and result in the reduction of mosquito density. The high fitness cost of this multiple resistance on larval mortality and/or the time of development of immature stages of resistant mosquitoes suggests that a resistance management strategy implemented before the allele becomes fixed in the population could effectively reduce P450-mediated metabolic resistance in the field.

### 4.3. Association between the 6.5kb SV Resistance Marker and Female Longevity

Adult longevity of female mosquitoes is one of the key features of vectorial capacity, which plays a major role in the transmission of the parasite. For example, in the case of *malaria,* the extrinsic incubation period for *Plasmodium* is approximately two weeks depending on the environmental conditions, and females must survive this period for transmission of the parasite. In our findings, the longevity of the female *An. funestus* was not impacted by the *6.5kb-SV-R* resistant allele. In contrast to this observation, Okoye et al. reported that resistant females of *An. funestus* had a longer life span than the susceptible strains [31]. The difference observed in their study was probably due to the fact that the resistant and the susceptible strains had different genetic backgrounds and the crossing between both strains as we did in this study can eliminate the difference. Rivero et al. reported that GSTs are known to protect mosquitoes against oxidative stress, which results in increased longevity, whereas the increased activity of monoxygenases is associated with increased oxidative stress in mosquitoes [42]. The increased oxidase stress due to overproduction of monoxygenases could, therefore, reduce the longevity of insects although no such impact was seen in this study. As observed in our study, Rowland also noticed no differences in the longevity of two dieldrin resistant malaria vectors *An. gambiae* and *An. stephensi* [33]. However, several studies reported reduced longevity of resistant mosquitoes compared to the susceptible [22,30,34,43]. Reduction in female longevity has the chance to reduce the gonotrophic cycles and the number of progenies, whereas the reduced longevity of males may lead to a reduction in mating opportunities. These factors could reduce the frequency of blood-feeding and pathogen diffusion in short-lived mosquitoes [44].

### 4.4. Restoration of Susceptibility

The frequency and the degree of dominance of resistant genes in a population is one of the factors that could influence the intensity and the development of physiological resistance in a population [45]. Knowledge of the reversal rate for insecticides such as pyrethroids is therefore crucial before implementing any resistance management strategy in the field. In this study, a significant decrease in the frequency of the *6.5kb-SV*_R resistant allele was observed after ten generations in the insecticide-free environment. This reduction in the resistant allele frequency could be attributed either to the accumulation of deleterious effects observed in some life traits of the vector as noticed for fecundity and larval development here [46] or to the pleiotropic effect of other genes very close to *CYP6P9a*. Mating, copulation, and insemination efficiency are other key factors that were not assessed in this study but which could have contributed to the reversal observed since female anopheles are inseminated only once during their lifespan.

## 5. Conclusions

This study investigating the fitness cost of the structural variant between the duplicated P450-based metabolic resistance to pyrethroids in *An. funestus* revealed that the *6.5kb-SV*_R resistance allele combines with both genes to significantly exacerbate the fitness cost on fecundity, fertility, and the larval development of resistant mosquitoes. The negative impact of insecticide resistance on different fitness cost parameters is very important against the maintenance and the dispersion of resistant individuals in the field. This should encourage future strategies based on the rotation of insecticides to reduce the selection pressure and to slow the spread of pyrethroid resistance.

## Data Availability

Data supporting the conclusions of this article are included within the article and its additional files.

## Supplementary Materials

The following supporting information can be downloaded at: https://www.mdpi.com/article/10.3390/genes13040626/s1, Figure S1: Schematic representation of the *6.5-kb* insertion in FUMOZ-R in comparison to FANG (Mugenzi et al. 2020) [26]. (a,b); result of pool-seq and (c) genomic location of the *6.5kb-sv*. Table S1: Variation in the *6.5kb-SV* genotypes and allele frequency for ten generations in the insecticide free-environment.
