# Supplementary material for: A 6.5kb Intergenic Structural Variation Exacerbates the Fitness Cost of P450-Based Metabolic Resistance in the Major African Malaria Vector Anopheles funestus
Source: Genes (Basel). 2022 Apr 1;13(4):626. doi: 10.3390/genes13040626 (PMC9025909; doi:10.3390/genes13040626)
Supplement: Supplementary file 1 [file genes-13-00626-s001.zip › Table S1.pdf]

Table S1: variation in the *6.5kb-SV* genotypes and allele's frequency for ten generations in the insecticides free-environment

	Generations									
	**F1**	**F2**	**F3**	**F4**	**F5**	**F6**	**F7**	**F8**	**F9**	**F10**
	**Genotypes**									
***6.5kb-SV*_RR**	00 (0%)	02 (10%)	06 (15%)	18 (12%)	09 (11%)	10 (10%)	05 (09%)	11 (12%)	05 (11%)	05 (11%)
***6.5kb-SV*_RS**	45 (100%)	14 (70%)	20 (50%)	67 (47%)	40 (47%)	48 (48%)	26 (47%)	44 (47%)	19 (43%)	13 (30%)
***6.5kb-SV*_SS**	00 (0%)	04 (20%)	14 (35%)	58 (41%)	36 (42%)	41 (42%)	24 (43%)	38 (41%)	20 (45%)	25 (59%)
**Total**	**45**	**20**	**40**	**137**	**85**	**99**	**55**	**93**	**44**	**42**
	**Alleles**									
***6.5kb-SV*_R**	0.50	0.45	0.40	0.36	0.34	0.34	0.33	0.35	0.33	0.27
***6.5kb-SV*_S**	0.50	0.55	0.60	0.64	0.66	0.66	0.67	0.65	0.67	0.73
**Total**	**1**	**1**	**1**	**1**	**1**	**1**	**1**	**1**	**1**	**1**
