# Supplementary figures and images for: A 6.5kb Intergenic Structural Variation Exacerbates the Fitness Cost of P450-Based Metabolic Resistance in the Major African Malaria Vector Anopheles funestus
Source: Genes (Basel). 2022 Apr 1;13(4):626. doi: 10.3390/genes13040626 (PMC9025909; doi:10.3390/genes13040626)

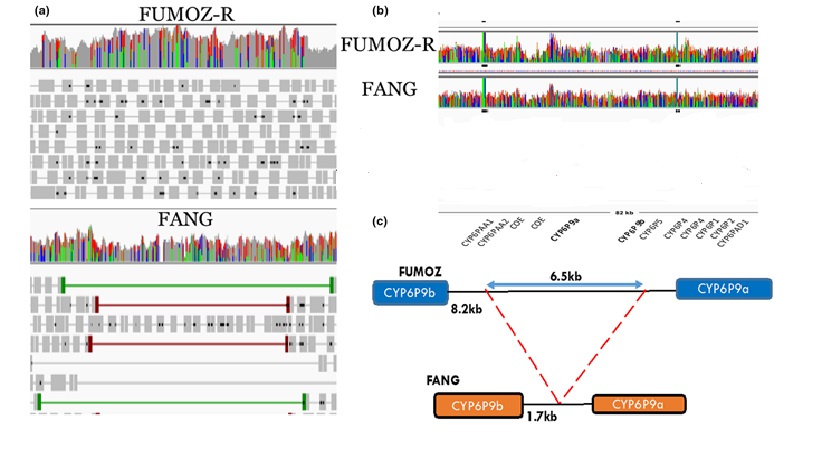

Supplement: Supplementary file 1 [file genes-13-00626-s001.zip › Suppl Figure S1.jpg]
